# An Atypical Presentation of Spontaneous Coronary Artery Dissection

**DOI:** 10.7759/cureus.8538

**Published:** 2020-06-09

**Authors:** Neil P Larson, Rachel E Bridwell, Lloyd Tannenbaum

**Affiliations:** 1 Emergency Medicine, Brooke Army Medical Center, Fort Sam Houston, San Antonio, USA; 2 Emergency Medicine, Brooke Army Medical Center, Fort Sam Houston, USA

**Keywords:** spontaneous coronary artery dissection, acute coronary syndrome

## Abstract

Spontaneous coronary artery dissection is a rare form of acute coronary syndrome (ACS) resulting from tears in the coronary vessel lumen leading to myocardial ischemia. Historically, younger to middle-aged Caucasian females without traditional risk factors for ACS are most commonly affected. The authors present the case of an African American female with numerous traditional ACS risk factors who presented to the emergency department with chest pain. Her workup revealed a stable electrocardiogram despite multiple sets of serially elevating cardiac enzymes and the patient was ultimately diagnosed with a spontaneous coronary artery dissection.

## Introduction

Spontaneous coronary artery dissection (SCAD), a tearing of the intimal layer of the coronary artery, and the subsequent luminal hematoma formation is a rare cause of myocardial ischemia, accounting for only 0.4% of all forms of acute coronary syndrome (ACS) [1]. Females are predominantly affected by SCAD, contributing up to 25%-35% of cases of ACS in those less than 50 years of age [2-4]. More than 80% of those affected are Caucasian, and those without traditional ACS risk factors are at higher risk [2-4]. Peripartum states have been associated with SCAD, with hormonal fluctuations theorized to alter vessel structure [3]. Other reported conditions associated with SCAD include a multitude of inflammatory and connective tissue disorders [4].

## Case presentation

**Figure 1:**
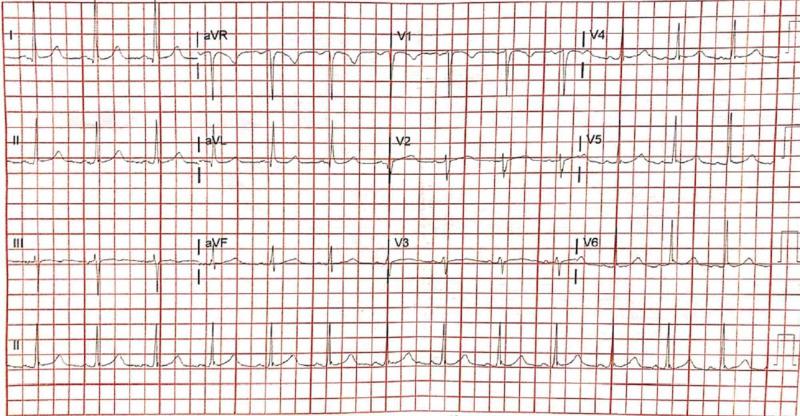
Initial electrocardiogram demonstrating a normal sinus rhythm.

An obese, African American, 44-year-old female with a past medical history of hypertension and hyperlipidemia presented to the emergency department (ED) with a chief complaint of sharp, central chest pain radiating to the left upper extremity that awoke her from sleep. The patient arrived at the hospital within 30 minutes of symptom onset, with initial vital signs of blood pressure 172/86 mm Hg, heart rate 91 beats per minute, respiratory rate of 16 breaths per minute, oxygen saturation of 99% on room air and pain level of 9/10. Electrocardiogram (ECG) performed in triage was normal sinus rhythm without evidence of ischemia (Figure 1). Initial labs revealed troponin elevated at 0.11 ng/mL (normal range 0.0-0.03 ng/mL); other lab values including those reflected in the complete blood count, complete metabolic panel, coagulation panel and human chorionic gonadotropin were within normal limits. Chewable aspirin, 324 mg, was immediately administered; 975 mg of oral acetaminophen, and a total of 1.2 mg of sublingual nitroglycerin over three doses were administered for analgesia, which reduced the patient’s chest pain to 3/10.

**Figure 2:**
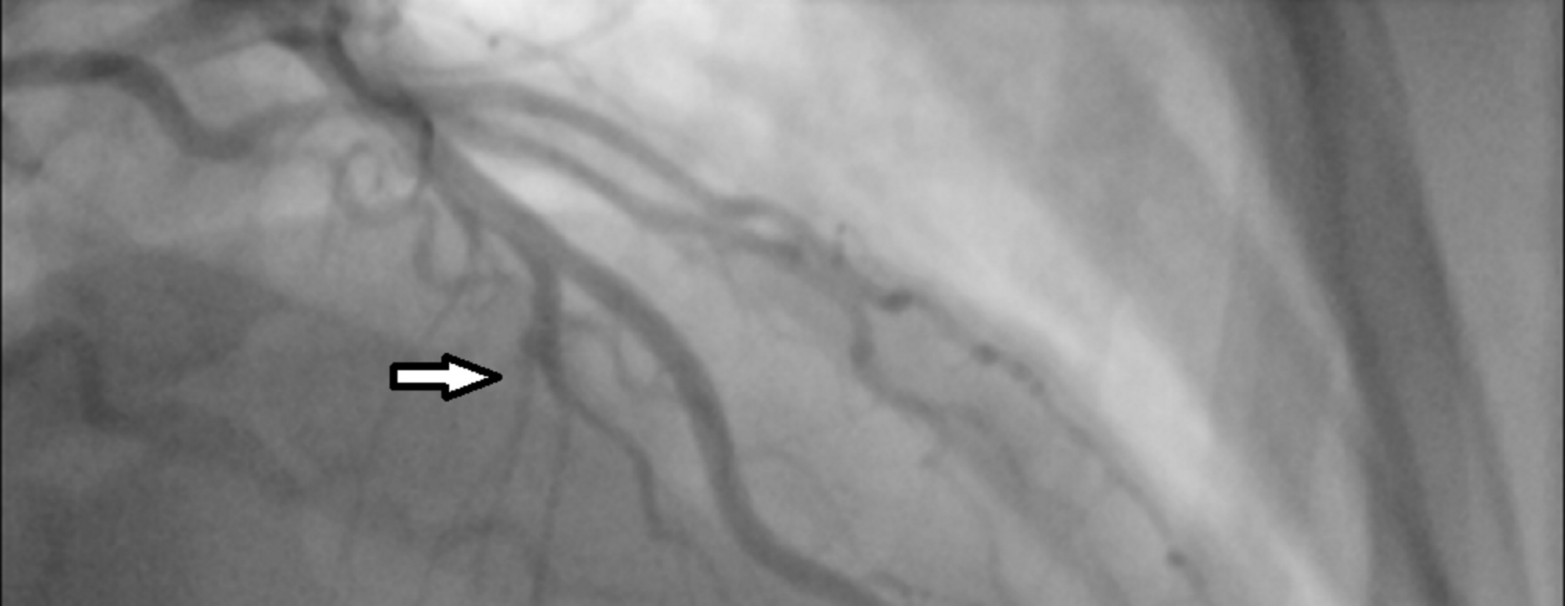
Obtuse marginal 3 branch of the lateral circumflex artery demonstrating a tortuous trajectory with narrowing and abrupt progression from normal to abnormal artery anatomy (white arrow), suggestive of vessel dissection.

Posterioranterior and lateral chest plain films were without radiographic evidence of acute process. Two additional ECGs, which were performed within the subsequent 1 hour and 40 minutes of the initial study, remained unchanged, and repeat troponin demonstrated escalation to 0.59 ng/mL. Anticoagulation therapy with enoxaparin was administered subcutaneously at 1 mg/kg, and a diagnosis of non-ST elevation myocardial infarction (NSTEMI) was established. After cardiology consultation, the patient was admitted to the cardiac care unit. The patient underwent subsequent left and right heart cardiac catheterizations that revealed findings consistent with coronary artery dissection of the obtuse marginal 3 (OM3) branch of the left circumflex artery (Figure 2). No underlying atherosclerosis was identified during the study. The remainder of the inpatient course was uneventful, and the patient was placed on daily oral 81 mg aspirin and her antihypertensive regimen was also optimized. While dual antiplatelet therapy was the original therapeutic plan, the patient was unable to tolerate clopidogrel due to a multisystem drug reaction, and it was unknown whether alternatives such as ticagrelor or prasugrel were considered. Outpatient CT angiography studies and rheumatology referral were ordered as part of the investigation for extracoronary vessel pathology including vessel narrowing and aneurysms that are often found in comorbid connective tissue conditions including fibromuscular dysplasia (FMD). Cardiac rehabilitation and short-term cardiology appointments were also scheduled. The patient continued to do well with no return to the ED within the next 30 days. The patient was ultimately not diagnosed with other comorbid vascular lesions or connective tissue disease including FMD.

## Discussion

Although SCAD is an uncommon form of ACS, the most common presenting symptom of SCAD is chest discomfort [5]. While studies vary widely with reported ECG findings in SCAD, the majority of patients present with ST changes reflective of ischemia either in the form of ST elevations, depressions or T-wave inversions, but nonspecific ST changes and normal ST segments are also reported [4]. In contrast, cardiac enzymes are universally elevated [4]. While our patient did not have a dissection of a larger vessel that may have led to ST and T changes reflective of ischemia, as with the patient's ethnicity and risk factors, this further convoluted the initial clinical picture that would otherwise represent a more common SCAD patient. As SCAD presents indistinguishable from other forms of ACS, it should be treated as such in the ED with standard treatments including aspirin, consideration of anticoagulation and antiplatelet therapy in conjunction with emergent cardiology consultation. Though forms of cardiac imaging such as coronary computed tomography angiography have been utilized in SCAD diagnosis, reassuring CT results do not rule out SCAD diagnoses, especially in smaller and distal coronary vessels, and as with other forms of ACS, coronary angiography remains the definitive diagnostic modality of SCAD [3,6]. Furthermore, optical coherence tomography and intravenous ultrasonography may also both aid in the diagnosis of SCAD when angiography does not yield conclusive results [3]. However, these studies are largely supplementary as definitive evidence on coronary angiography is sufficient to make the diagnosis. The left anterior descending artery is the most frequently affected coronary artery; however, any may be affected [3,4]. Again, here the patient fell into an anatomical minority with dissection of the OM3 branch of the left lateral circumflex artery dissection. As percutaneous coronary intervention has been associated with complications and poor outcomes, conservative management is the optimal treatment of SCAD patients, with repeat angiography demonstrating healing of SCAD lesions [1,3,4]. However, up to 10% of patients may experience complications including extension of the existing dissection or new dissection during the inpatient course [3]. Though no consensus on standard medical therapy after SCAD diagnosis has been established, long-term daily aspirin use is often recommended [3,7]. Dual antiplatelet therapy is a potential consideration in select patients; however, consistent studies again are lacking for universal support. While a multitude of inflammatory and connective tissue disorders have been linked to SCAD, FMD has a particularly strong association, with one recent study demonstrating 86% of SCAD patients with this diagnosis upon subsequent workup [8]. Accordingly, in patients diagnosed with SCAD, FMD workup is strongly advocated for detection and any needed intervention for extracoronary lesions and aneurysms [3,4].

## Conclusions

The authors presented the case of SCAD that varied from the traditional patient race, risk factors, electrocardiographic findings and vessel location. SCAD is a rare but an increasingly recognized form of cardiac ischemia, most commonly diagnosed in young females without traditional risk factors for ACS. Definitive diagnosis usually requires coronary angiography, as the acute presentation and initial workup may mimic the statistically more common forms of atherosclerotic ACS. As such, patients with this suspected diagnosis should still be treated emergently accordingly in conjunction with cardiology consultation. Underlying vascular phenomena including FMD are commonly associated with SCAD and warrant further workup.
